# Protein–Protein Interactions of Seryl-tRNA Synthetases with Emphasis on Human Counterparts and Their Connection to Health and Disease

**DOI:** 10.3390/life14010124

**Published:** 2024-01-15

**Authors:** Morana Dulic, Vlatka Godinic-Mikulcic, Mario Kekez, Valentina Evic, Jasmina Rokov-Plavec

**Affiliations:** Division of Biochemistry, Department of Chemistry, Faculty of Science, University of Zagreb, 10000 Zagreb, Croatia; mdulic@chem.pmf.hr (M.D.); vgodinic@lzmk.hr (V.G.-M.); mario.kekez@aandb.hr (M.K.); vevic@chem.pmf.hr (V.E.)

**Keywords:** aminoacyl-tRNA synthetase, seryl-tRNA synthetase, *SARS1*, translation, tRNA channeling, protein–protein interaction, VEGFA, angiogenesis, senescence, telomere

## Abstract

Seryl-tRNA synthetases (SerRSs), members of the aminoacyl-tRNA synthetase family, interact with diverse proteins, enabling SerRSs to enhance their role in the translation of the genetic message or to perform alternative functions in cellular processes beyond translation. Atypical archaeal SerRS interacts with arginyl-tRNA synthetase and proteins of the ribosomal P-stalk to optimize translation through tRNA channeling. The complex between yeast SerRS and peroxin Pex21p provides a connection between translation and peroxisome function. The partnership between *Arabidopsis* SerRS and BEN1 indicates a link between translation and brassinosteroid metabolism and may be relevant in plant stress response mechanisms. In *Drosophila*, the unusual heterodimeric mitochondrial SerRS coordinates mitochondrial translation and replication via interaction with LON protease. Evolutionarily conserved interactions of yeast and human SerRSs with m^3^C32 tRNA methyltransferases indicate coordination between tRNA modification and aminoacylation in the cytosol and mitochondria. Human cytosolic SerRS is a cellular hub protein connecting translation to vascular development, angiogenesis, lipogenesis, and telomere maintenance. When translocated to the nucleus, SerRS acts as a master negative regulator of *VEGFA* gene expression. SerRS alone or in complex with YY1 and SIRT2 competes with activating transcription factors NFκB1 and c-Myc, resulting in balanced *VEGFA* expression important for proper vascular development and angiogenesis. In hypoxia, SerRS phosphorylation diminishes its binding to the *VEGFA* promoter, while the lack of nutrients triggers SerRS glycosylation, reducing its nuclear localization. Additionally, SerRS binds telomeric DNA and cooperates with the shelterin protein POT1 to regulate telomere length and cellular senescence. As an antitumor and antiangiogenic factor, human cytosolic SerRS appears to be a promising drug target and therapeutic agent for treating cancer, cardiovascular diseases, and possibly obesity and aging.

## 1. The Importance of Aminoacyl tRNA-Synthetases

Aminoacyl-tRNA synthetases (aaRSs) are ancient ubiquitous enzymes present in every living cell. They catalyze the aminoacylation reaction by binding the cognate amino acid to the appropriate tRNA molecule, thus preparing the foundation for protein biosynthesis. The aminoacylation reaction proceeds in two steps. In the first step, the α-carboxylate group of the amino acid substrate attacks the α-phosphate of ATP, resulting in the formation of aminoacyl-adenylate and inorganic pyrophosphate. In the second step, the 2′- or 3′-hydroxyl group at the 3′-end of the tRNA molecule nucleophilically attacks the carbonyl carbon atom of aminoacyl-adenylate (aa-AMP), forming aminoacyl-tRNA (aa-tRNA) and AMP [1,2,3]. aa-tRNA is used by the ribosome as a substrate for translation of the genetic message into polypeptides. aaRS specific for an individual amino acid is abbreviated with the three-letter code for the amino acid, e.g., SerRS for seryl-tRNA synthetase.

In non-photosynthetic eukaryotic organisms (most lower eukaryotes, metazoa), there are two protein-synthesizing compartments: the cytosol and mitochondria. Generally, for each aaRS, there are two separate nuclear genes encoding cytosolic and mitochondrial aaRSs. Rarely, one nuclear gene encodes both the cytosolic and mitochondrial isoforms through alternative splicing, transcription, or translational initiation [4,5]. Mitochondrial aaRSs are synthesized in the cytosol and imported into mitochondria. Although photosynthetic eukaryotic organisms (plants) contain a third protein synthesizing compartment (chloroplasts), these organisms also contain two separate nuclear genes encoding two aaRSs; one is always dually targeted, either to the cytosol and mitochondria or to mitochondria and chloroplasts [4,6,7]. Additionally, several cytosolic aaRSs are also localized in the nucleus of cells from several species [8].

Due to their essential role in protein biosynthesis, the appearance of aaRSs occurred very early in the evolutionary past. As part of further evolutionary development, the catalytic core of aaRS was structurally enriched with additional domains and motifs [9]. These newly added components broadened aaRS functionality by enabling better recognition of cognate tRNA molecules [10] and proofreading of mischarged tRNAs [11]. Beyond translation, these additional domains contributed to the non-canonical functions of aaRS in gene regulation, cell signaling, inflammation, angiogenesis, tumorigenesis, immune processes, and RNA viral infections [12,13,14,15,16,17,18].

Research has revealed an important role of multiple aaRSs in pathology and their potential use as pharmacological targets and therapeutic reagents [19,20,21]. Disease-associated mutations in cytoplasmic aaRSs are mainly associated with Charcot-Marie-Tooth and related neuropathies, while mutations in mitochondrial aaRSs are associated with a wider variety of syndromes and diseases, which include a large spectrum of clinical manifestations, particularly muscular and neurological disorders [22,23,24]. Anti-synthetase syndrome is a rare autoimmune inflammatory myopathy that involves the production of autoantibodies that disrupt aaRS proteins [25]. Genetic and postgenetic abnormalities of human aaRSs are associated with cancer development, progression, and survival through their nonconventional and catalytic activities [17,26].

## 2. Protein–Protein Interactions of aaRSs

AaRSs are capable of associating with proteins into higher order complexes engaged in a variety of cellular tasks in all three domains of life [13,17,27,28,29,30,31]. The role of aaRS interactions with other proteins is dual: (a) optimization of translational functionality by promoting aaRS aminoacylation activity and tRNA channeling to the ribosome, (b) manifestation of various non-canonical functions beyond translation. Several examples of protein–protein interactions of aaRSs in higher vertebrates are given below.

In mammalian cells, several aaRSs coexist in a multi-synthetase complex (MSC) composed of 12 proteins (nine aaRS: LeuRS, IleRS, LysRS, GluProRS, MetRS, GlnRS, ArgRS, AspRS, and three aaRS-interacting multifunctional proteins (AIMPs): AIMP1, AIMP2, AIMP3) which participate in tRNA binding, complex stabilization, and non-canonical actions [26,32]. Environmental cues, resulting in posttranslational modifications, mediate the release of aaRSs from the MSC, enabling their non-canonical functions. For example, during an inflammatory response, the interferon-γ (IFN-γ) stimulates phosphorylation of GluProRS, which leads to its release from MSC and the formation of the heterotetrameric IFN-γ-activated inhibitor of translation (GAIT) complex, including NS1-associated protein 1 (NSAP1), ribosomal protein L13a, and glyceraldehyde 3-phosphate dehydrogenase (GAPDH). The newly formed complex binds to the structural GAIT element in the 3′ UTRs of multiple inflammation-related mRNAs leading to their translational silencing and modulation of the inflammatory process [33,34]. The GluProRS interactions with the tRNA-dihydrouridine synthase 2 (DUS2) in lung cancer and with the SCY1-like protein 2 (SCYL2) in gastric cancer drive cell proliferation and have tumorigenic effects [35,36].

Upon laminin signaling, LysRS undergoes phosphorylation, which leads to its release from MSC and translocation to the plasma membrane. In the membrane, LysRS binds laminin receptor 67LR, disturbing its ubiquitin-dependent degradation and increasing laminin-induced cell migration [37]. Upon antigen activation in mast cell, phosphorylated LysRS translocates to the nucleus, where it binds a complex of microphthalmia transcription factor (MITF) and its inhibitor, the histidine triad nucleotide-binding protein 1 (Hint1). LysRS produces the secondary messenger Ap4A, which interacts with Hint1, hindering its association with MITF. Consequently, MITF promotes the expression of target genes that regulate the immune response [38]. LysRS extracellular secretion is mediated by the caspase-8 cleavage of the LysRS N-terminus. The syntenin involved in exosome secretion interacts with the C-terminal part of the LysRS and directs it towards exosomes and out of the cell to trigger an inflammatory response [39].

Mammalian GlyRS is a freestanding aaRS involved in the inhibition of the extracellular signal-regulated kinase (ERK) signaling pathway important for cancer survival. When macrophages are stimulated by the tumor-derived Fas ligand, they secrete GlyRS, which then binds to the cadherin 6 protein on cancer cells, leading to the release of phosphatase 2A, a deactivator of the ERK signaling pathway [40].

The unique N-terminal extension of vertebrate ThrRS enables the enzyme to recruit eukaryotic translation initiation factor 4E family member 2 (eIF4E2) and form a novel translation initiation complex [41]. This machinery positively regulates the translation initiation of mRNAs required for vertebrate development.

The TyrRS of higher eukaryotes contains an additional domain at its C-terminus that shares 49% sequence similarity with the pro-inflammatory cytokine endothelial monocyte-activating protein II (EMAP II) [42]. During cell apoptosis, TyrRS is secreted extracellularly by endothelial cells and cleaved by the leukocyte elastase, whereby the part with a C-terminal EMAPII-like domain causes chemotaxis of leukocytes and stimulates the release of tumor necrosis factor-α and tissue factor. The remaining N-terminal fragment interacts with C-X-C chemokine receptor type CXCR1/2, exhibiting pro-inflammatory chemotactic and pro-angiogenic effects [43].

The antagonistic angiostatic action was observed with mini-TrpRS, an isoform of human TrpRS created by alternative mRNA splicing or proteolytic cleavage of the full-length TrpRS under the influence of IFN-γ [44]. Extracellular mini-TrpRS binds to the adhesion protein vascular endothelial cadherin on endothelial cells, which inhibits angiogenesis by decreasing the activation of the ERK signaling pathway [45].

## 3. Seryl-tRNA Synthetases

The canonical activity of SerRS is aminoacylation of tRNA^Ser^ with serine [46,47]. In most organisms, SerRS additionally aminoacylates selenocysteine-specific tRNA (tRNA^Sec^) with serine, which is the first step in the metabolic pathway for translational incorporation of selenocysteine into selenoproteins [48,49]. Recently, a novel mechanism involving SerRS was described for the regulation of the physiological translational read-through of specific, non-selenocysteine, mRNAs [50].

**Figure 1:**
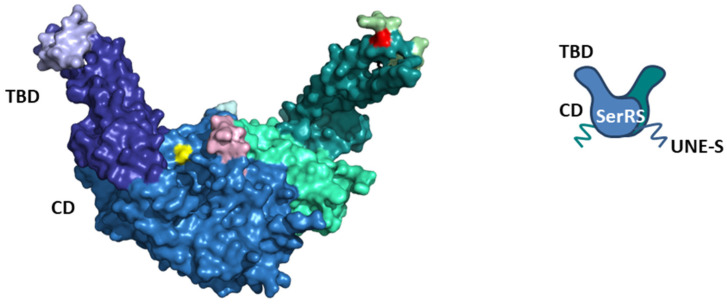
Structural representation of human cytosolic SerRS ((**left**), surface view; (**right**), schematic view). The surface view represents the structure of human cytosolic SerRS in complex with tRNA^Sec^ (PDB: 4RQE, tRNA is not shown). Subunit 1 is colored as follows: catalytic domain (CD) in sky blue, tRNA-binding domain (TBD) in deep blue, higher eukaryote-specific insertion I in light blue, higher eukaryote-specific insertion II in light cyan, and Ser241 in yellow (Ser101 is not visible). Subunit 2 is colored as follows: CD in green cyan, TBD in deep teal, insertion I in light green, insertion II in light pink, and Ser101 in red (Ser241 is not visible). The C-terminal vertebrate-specific UNE-S domain is flexible and not visible in the structure, but is depicted in the schematic view. Serines 101 and 241 are sites of posttranslational modifications. Ser101 is phosphorylated during hypoxia and glycosylated during starvation, while Ser241 is phosphorylated during hypoxia.

SerRS is a homodimeric enzyme. Each monomer is composed of the N-terminal tRNA-binding domain (TBD) and the globular C-terminal catalytic domain (CD) that is also responsible for dimerization (Figure 1) [47]. The CD consists of a seven-stranded β-sheet flanked by α-helices and contains three conserved motifs: motif 1 takes part in forming a dimer interface, while motifs 2 and 3 play a role in binding the ATP, amino acid, and 3′-end of tRNA [51]. The N-terminal TBD features an antiparallel coiled coil composed of two long α-helices, protruding away from CD. TBD recognizes the long variable arm of tRNA^Ser^ [52,53] and directs the acceptor arm of tRNA^Ser^ into the active site of the catalytic domain in the opposite subunit [54,55]. Therefore, tRNA^Ser^ binds across both subunits. Animal mitochondrial SerRSs have evolved a unique dual mode for the recognition of two highly divergent mitochondrial tRNAs^Ser^ that do not possess a long variable arm typical for tRNAs^Ser^ [56,57]. Interestingly, in the mitochondria of protostome animals, SerRS is a heterodimer, where one subunit lacks TBD, while the other is catalytically inactive [58]. Another exception to the standard SerRS structure is the atypical non-canonical SerRS from methanogenic archaea, which contains Zn^2+^ in the active site involved in serine binding and significantly larger TBD with a completely different and unrelated fold compared to the conventional SerRS found in all three domains of life [59,60].

Eukaryotic cytosolic SerRSs have idiosyncratic C-terminal extensions rich in basic amino acids that are not present in their prokaryotic counterparts [61]. In yeast, the SerRS C-terminal extension is important for interaction with the protein Pex21p [61,62]. Although the lysine-rich C-terminal extension in the plant *Arabidopsis thaliana* SerRS resembles the bipartite nuclear localization signal (NLS), the protein is exclusively localized in the cytosol [6]. The C-terminal extension specific for vertebrate SerRSs, the unique domain UNE-S, harbors robust NLS that redirects the protein from the cytosol into the nucleus, where it exhibits non-canonical functions unrelated to translation [63]. Interestingly, the SerRS from the fungus *Trametes hirsuta* also shows nuclear localization and acts as a transcriptional repressor [64]. An evolutionary addition to higher eukaryote cytosolic SerRSs are two insertions (Figure 1). Insertion I in TBD mediates long-range conformational and functional communication with the active site, while Insertion II in CD, although in proximity to the active site, plays a negligible role in aminoacylation [65].

Crystal structures of cytosolic SerRSs from humans and plant *A. thaliana* revealed a disulfide bond in each subunit, which is unusual, considering that cytosolic proteins in general do not possess disulfide bonds due to the reducing nature of the cytosol [66,67]. The disulfide bond formed in oxidative conditions restricts the flexibility of TBD and consequently the aminoacylation activity of human SerRS, which may trigger signaling pathways as a response to oxidative damage [66]. The cysteines involved in the disulfide bond in *A. thaliana* SerRS are evolutionarily conserved in all green plants. In oxidizing conditions, the formation of a disulfide link did not abolish the activity of plant SerRS, compared to the severely impaired activity of cysteine-to-serine mutants, which may be beneficial for translation during oxidative stress conditions in plants [68].

Human SerRSs are linked to rare hereditary diseases and disorders. Pathogenic variants in the *SARS1* gene encoding cytosolic SerRS have been implicated in neurodevelopmental, neurological, and movement disorders with various symptoms [69,70,71,72,73]. Several mutations in the *SARS2* gene encoding mitochondrial SerRS have been associated with conditions such as HUPRA (hyperuricemia, pulmonary hypertension, renal failure in infancy, and alkalosis) syndrome and progressive spastic paresis [74,75,76,77,78,79]. Transcriptomic and proteomic data revealed aberrant cytosolic and mitochondrial SerRS mRNA and protein levels, depending on the cancer type [17].

**Figure 2:**
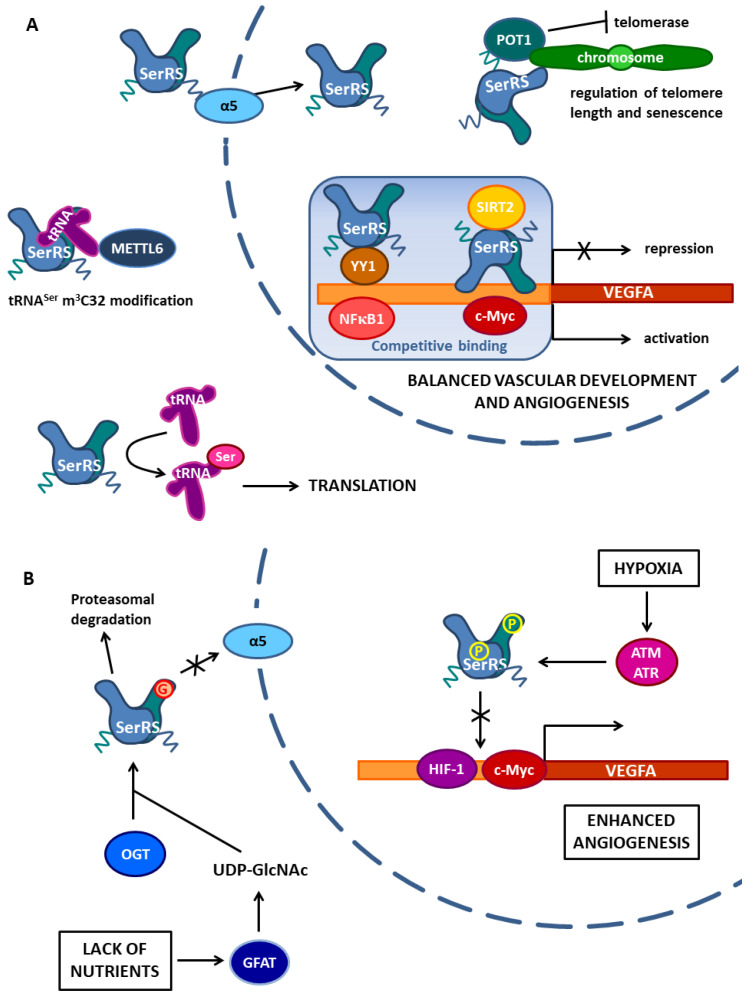
Non-canonical functions of human cytosolic SerRS enabled by protein–protein interactions. (**A**) Human cytosolic SerRS via nuclear localization signal in the UNE-S domain interacts with α5 importin, which enables its translocation to the nucleus. In the nucleus, SerRS regulates *VEGFA* expression by competitive binding in the *VEGFA* promoter region. SerRS competes with transcriptional activator c-Myc at the proximal site and recruits SIRT2 deacetylase to erase histone acetylation. Furthermore, SerRS binds to the YY1 protein and the complex competes with transcriptional activator NFκB1 for binding at the distal part of the *VEGFA* promoter. SerRS binds both SIRT2 and YY1 through its catalytic domain. The balance between *VEGFA* transcription activation and repression enables normal vascular development and angiogenesis. Additionally, in the nucleus, SerRS binds to telomeric repeats and, using the UNE-S domain, interacts with Sheltering protein POT1, enhancing its tethering to telomeres. This interaction regulates telomere length and senescence by inhibiting telomerase. In the cytosol, SerRS indirectly binds methyltransferase METTL6, enabling its tRNA^Ser^ m^3^C32 modification activity. The complex is formed via tRNA^Ser^, which binds the SerRS tRNA-binding domain with its variable arm and METTL6 with its anticodon loop. The canonical function of SerRS in translation is also shown. (**B**) Protein–protein interactions of human cytosolic SerRS depend on stress-induced posttranslational modifications. Under hypoxic conditions, SerRS is phosphorylated on Ser101 and Ser241 by ATM/ATR kinases in the nucleus. Phosphorylations reduce the SerRS binding capacity for DNA and therefore c-Myc and HIF-1, the transcription factor induced in hypoxia, bind the *VEGFA* promoter, and activate *VEGFA* expression, which leads to enhanced angiogenesis. In the cytosol, SerRS responds to the lack of nutrients. Starvation in tumor cells causes the production of extracellular vesicles containing GFAT (glutamine fructose-6-phosphate amidotransferase), which enters the cytosol of endothelial cells and changes the glucose metabolism towards the production of UDP-GlcNAc. GlcNAc is transferred to Ser101 on SerRS by OGT (*O*-GlcNAc transferase), which binds the SerRS tRNA-binding domain. Glycosylated SerRS cannot interact with α5 importin to enter the nucleus and is also prone to proteasomal degradation. The lack of SerRS in the nucleus leads to the activation of *VEGFA* expression and angiogenesis.

**Figure 3:**
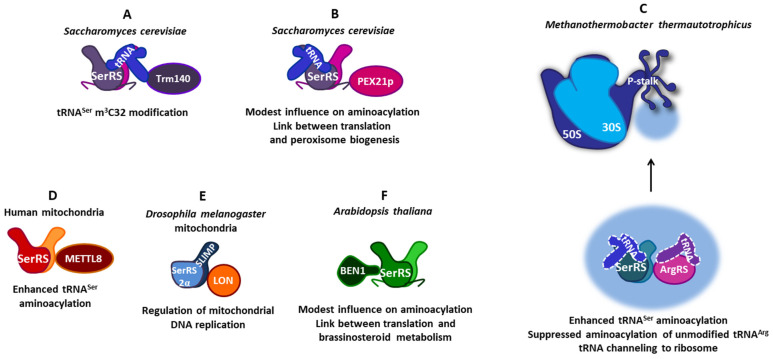
The versatility of protein–protein interactions of SerRSs from various organisms. (**A**) In the yeast *Saccharomyces cerevisiae*, SerRS forms a ternary complex with tRNA^Ser^ and Trm140 methyltransferase, enabling tRNA^Ser^ m^3^C32 modification. (**B**) Additionally, in *S. cerevisiae* SerRS, the C-terminal extension binds peroxin Pex21. This interaction modestly increases tRNA^Ser^ aminoacylation and implicates a connection between translation and peroxisome biogenesis. (**C**) SerRS from the methanogenic archaea *Methanothermobacter thermautotrophicus* forms a complex network of protein–protein interactions. The SerRS:ArgRS complex enhances tRNA^Ser^ aminoacylation and suppresses the aminoacylation of unmodified tRNA^Arg^. The association of both aaRSs with ribosomal proteins of the P-stalk of the large ribosomal subunit implicates tRNA channeling. (**D**) In human mitochondria, the interaction of SerRS with methyltransferase METTL8 slightly stimulates tRNA m^3^C32 modification and enhances tRNA^Ser^ aminoacylation. (**E**) In the fruit fly *Drosophila melanogaster* mitochondria, heterodimeric SerRS2 is composed of SerRS2α lacking a tRNA-binding domain and SerRS paralogue SLIMP with a defunct catalytic site. The interaction of SerRS2 with LON protease via SLIMP coordinates mitochondrial translation and replication. (**F**) In the plant *Arabidopsis thaliana*, the SerRS catalytic domain interacts with the N-terminal domain of BEN1 oxidoreductase, which modestly influences aminoacylation and suggests a link between translation and brassinosteroid metabolism.

SerRSs from various organisms interact with diverse proteins, enabling SerRS to enhance its role in the translation of the genetic message or to perform alternative functions in cellular processes beyond translation (Figure 2 and Figure 3). In this review, we describe the versatile protein–protein interactions of SerRSs, with an emphasis on human SerRS-containing protein complexes and their connection to health and disease.

## 4. Vertebrate SerRS Localized in the Nucleus Acts as a Transcriptional Repressor of *VEGFA* Gene Expression

Two independent forward-genetic studies in zebrafish showed that cytosolic SerRS is important in vascular development by preventing vascular over-expansion and that this essential function is independent of its canonical aminoacylation activity [80,81]. The vascular role of SerRS is conserved between zebrafish and humans, and this non-canonical activity of SerRS has been linked to attenuating the expression of *VEGFA*, the gene encoding vascular endothelial growth factor A (VEGFA), a key regulator of vasculogenesis and angiogenesis that plays pivotal roles in the establishment of the vascular network [82]. Among mutations that gave rise to vasculature abnormalities, two resulted in premature stop codons, eliminating a part of the aminoacylation domain and the entire UNE-S domain, which in turn prevented the nuclear localization of SerRS [63]. The third was a missense mutation, located in the aminoacylation domain, which sequesters NLS in alternative conformation, resulting in inefficient translocation to the nucleus. Therefore, the consequences of all three mutations indicated that the non-canonical function of SerRS in vascular development depends on its localization in the nucleus and that the evolutionary acquisition of UNE-S has a role in the establishment of the closed circulatory systems in vertebrates.

### 4.1. SerRS Competes with Transcriptional Activator c-Myc in the Proximal VEGFA Promoter Region and Recruits SIRT2 to Remove Histone Acetylation

Shi et al. elucidated the mechanism by which human cytosolic SerRS (encoded by the *SARS1* gene) localized in the nucleus controls *VEGFA* expression (Figure 2A) [83]. A chromatin immunoprecipitation (ChIP) experiment showed that SerRS binds to the proximal *VEGFA* promoter in the region from −1.5 kb to +1 of the start site. Further dissection of SerRS binding identified the 21 nt fragment (positions −59~−38 of the *VEGFA* gene) as the minimal binding site of SerRS. Interestingly, this site overlapped with the binding site of the complex between c-Myc and Myc-associated factor X (MAX), and c-Myc is a well-known transcriptional activator that plays a pivotal role in vascular development by promoting *VEGFA* expression [84]. Multiple domains of SerRS contributed to the interaction with DNA: motif V2-G14, loop T413-V420, two higher eukaryote-specific insertions (I and II) in TBD and CD, as well as UNE-S. Ectopic expression of SerRS repressed the overexpression of *VEGFA* driven by c-Myc, while knocking down endogenous SerRS expression resulted in a dramatic increase of endogenous c-Myc binding to the *VEGFA* promoter, indicating competition between c-Myc and SerRS for binding to the *VEGFA* promoter and the contrasting activity of c-Myc and SerRS in regulating *VEGFA* expression. Experiments in zebrafish showed that on the organism level, the balance of the opposing effects of SerRS and c-Myc on *VEGFA* expression is important for proper vasculature development [83].

c-Myc activates gene expression by recruiting histone acetyltransferases that modify histones and lead to open chromatin structures [85]. A large-scale protein–protein interaction study [86] implicated a putative interaction between SerRS and histone deacetylase sirtuin 2 (SIRT2) that regulates a broad range of processes, such as transcription, metabolism, neurodegeneration, and aging [87]. Co-immunoprecipitation and GST pull-down assays confirmed a direct interaction between SerRS and SIRT2 (Figure 2A) [83]. Mapping analysis showed that the CD of SerRS is responsible for the interaction with SIRT2, while the N- and C-terminal regions, outside the catalytic core of SIRT2, mediate the interaction with SerRS. SerRS promoted the deacetylase activity of SIRT2, and further experiments showed that nuclear SerRS acts to decrease the amount of acetylated histone H4 on the *VEGFA* promoter through the engagement of SIRT2 by SerRS. Disruption of SIRT2 by RNA interference (RNAi) completely reversed the transcriptional repression activity of SerRS on *VEGFA* expression, indicating that SIRT2 is a necessary cofactor for SerRS to repress *VEGFA* expression.

Therefore, an intricate balance between SerRS and c-Myc controls *VEGFA* expression through a tandem mechanism. By direct head-to-head competition, the SerRS localized in the nucleus inhibits the binding of c-Myc to the *VEGFA* proximal promoter. Additionally, DNA-bound SerRS recruits the SIRT2 histone deacetylase to epigenetically silence *VEGFA* expression by erasing histone acetylation, previously induced by c-Myc, and condensing the chromatin (Figure 2A) [83].

### 4.2. SerRS and YY1 Form a Transcriptional Repressor Complex Which Competes with Transcriptional Activator NFκB1 in the Distal VEGFA Promoter Region

Interestingly, another study showed that human cytosolic SerRS, when localized in the nucleus, is involved in the transcriptional regulation of *VEGFA* expression modulated by the distal upstream *cis*-regulatory elements (CRE) of the human *VEGFA* promoter (Figure 2A) [88]. Glutathione *S*-transferase (GST) pull-down coupled with liquid chromatography-tandem mass spectrometry (LC-MS/MS) and the subsequent co-immunoprecipitation (Co-IP) identified transcription factor Yin Yang 1 (YY1) as a protein interactor of SerRS. Domain mapping analysis revealed that the CD of SerRS is the key domain that interacts with YY1, similarly to the interaction between SerRS and SIRT2. A ChIP PCR experiment designed to scan the distal upstream sequences of the *VEGFA* gene indicated that region −4654 to −4623 was a critical segment for SerRS to regulate the *VEGFA* promoter activity. SerRS did not bind directly to the distal CRE element, but it did bind YY1, thereby increasing the affinity of YY1 for the −4654 to −4623 segment. Moreover, this segment could bind transcriptional activator nuclear factor NF-kappa-B 1 (NFκB1) and further experiments showed that the SerRS:YY1 complex competed with NFκB1 binding at the same segment. Another line of evidence indicated that the VEGFA protein level was decreased by adding either SerRS or YY1, but increased by adding NFκB1, suggesting a negative effector role of the SerRS:YYI complex in the context of human *VEGFA* promoter activity and a positive effector role for NFκB1. Experiments with zebrafish showed that blood vessel development in zebrafish embryos proliferated when the SerRS or YY1 genes were silenced, while it was inhibited by NFκB1 gene silencing. Importantly, the defects caused by the reduction of either SerRS or YY1 were rescued by an injection of NFκB1, or vice versa. In conclusion, without directly binding to the *VEGFA* distal promoter, SerRS interacts with YY1 to form a SerRS:YY1 repressor complex that competes with the positive effector NFκB1 for binding at the *VEGFA* distal promoter. Similarly to the ‘Yin-Yang’ relationship between SerRS and c-Myc [83], in this case, the balance between the SerRS:YY1 complex and NFκB1 modulates vascular development through controlling the production of VEGFA (Figure 2A) [88].

### 4.3. Hypoxia-Induced SerRS Phosphorylation Diminishes Its Binding to the VEGFA Promoter, Which Enhances VEGFA Expression and Angiogenesis

Reduced oxygen levels induce numerous molecular mechanisms that protect cells and tissues from ischemic injury [89]. Furthermore, oxygen depletion can lead to pathophysiological hypoxia, which can aid in the development of diseases such as cardiovascular disorders and solid tumors [90,91]. VEGFA is important during hypoxia responses because it stimulates angiogenesis [82]. The inactivation mechanism of the regulatory function of human SerRS, a negative regulator of *VEGFA* and angiogenesis during hypoxia, was recently discovered (Figure 2B) [92]. Under hypoxia, the expression of SerRS remained unchanged, but SerRS regulatory function was inactivated upon phosphorylation at Ser101 and Ser241 by ataxia telangiectasia-mutated (ATM) and ataxia telangiectasia-mutated and RAD3-related (ATR) kinases [92]. Both phosphorylation sites have conserved ATM/ATR substrate motifs in vertebrate SerRSs, concurrent with the important role of SerRS in vascular development and angiogenesis. ATM/ATR kinases participate in DNA damage response and are activated under hypoxic stress [93,94].

Phosphorylation of SerRS inhibited its transcription repressor activity, as shown in cell models or in vivo in zebrafish [92]. Knocking down either ATM or ATR or both kinases, as well as the addition of their inhibitors, greatly inhibited SerRS phosphorylation and consequently *VEGFA* expression. The nuclear localization of SerRS remained unchanged in hypoxia. In line with that, the nuclear distribution was similar for wild-type and SerRS mutant variants, where both phosphorylation sites (Ser101 and Ser241) were changed to either alanine (which prevents phosphorylation) or aspartate (which mimics phosphorylation). These mutants largely maintained the enzymatic activity of aminoacylation reaction. Furthermore, the interaction of SerRS with SIRT2 was not affected by hypoxia or mutations in phosphorylation sites. However, phosphor-mimicking mutations weakened SerRS interaction with the SerRS binding site in the proximal *VEGFA* promoter region. As phosphorylation sites S101 and S241 are in the vicinity of higher eukaryote-specific insertion I (G75-N97) in TBD and insertion II (G254-N261) in CD (Figure 1), which contribute to the binding of SerRS to DNA, the introduction of negative charges may lead to electrostatic repulsion between SerRS and DNA. Therefore, hypoxia and the subsequent phosphorylation of SerRS diminished its DNA binding capacity to the *VEGFA* proximal promoter and blocked the repression of *VEGFA* expression. Consequently, levels of c-Myc (which competes with SerRS for DNA binding) bound to the *VEGFA* promoter were increased during hypoxia. Interestingly, the amount of hypoxia-inducible factor 1 (HIF-1) bound to the *VEGFA* promoter was also increased. HIF-1 is a transcription factor specific for hypoxia response, which binds to the hypoxia-response element (HRE) in the promoter region of *VEGFA* and activates its expression [82]. Using SerRS phosphorylation-site mutants, it was shown that phosphorylation of SerRS was indeed a prerequisite for the hypoxia-induced removal of SerRS from DNA and the binding of c-Myc and HIF-1 to the *VEGFA* promoter [92]. On the other hand, the phosphorylation-deficient SerRS mutant prevented the hypoxia-induced binding of c-Myc and HIF-1 to the VEGFA promoter and the activation of *VEGFA* expression. Accordingly, this mutant strongly inhibited normal and tumor-derived angiogenesis in mice. Therefore, the posttranslational inactivation of SerRS, a negative regulator of angiogenesis, is a key step in the regulation of hypoxic angiogenesis and highlights the importance of nuclear SerRS in post-developmental angiogenesis in addition to vascular development (Figure 2B). Considering that nuclear SerRS inhibits both c-Myc and HIF-1, this may provide therapeutic opportunities to correct the dysregulation of angiogenesis in treating cancer and ischemic cardiovascular diseases [92].

### 4.4. Starvation-Induced SerRS Glycosylation Reduces Its Nuclear Localization Resulting in Enhanced VEGFA Expression and Angiogenesis

Cancer metabolic reprogramming is a set of changes that allows for rapid proliferation and enhances adaptability to external stimuli in tumor cells [95]. A recent study showed how metabolic programming promotes tumor angiogenesis through SerRS (Figure 2B) [96]. Bladder cancer cells activate the surrounding endothelial cells for angiogenesis by excreting small extracellular vesicles. Compared to the extracellular vesicles derived from normal tissue, the vesicles derived from tumor cells were enriched in glutamine fructose-6-phosphate amidotransferase (GFAT), a crucial enzyme of the hexosamine biosynthetic pathway that results in the synthesis of uridine diphosphate N-acetylglucosamine (UDP-GlcNAc), which is a substrate for *O*-linked β-N-acetylglucosamine protein modification (*O*-GlcNAcylation) performed by *O*-GlcNAc transferase (OGT) [96]. GFAT expression and secretion in the extracellular vesicles were induced by a lack of nutrients in the tumor’s microenvironment. The elevated concentration of GFAT in endothelial cells, which depended on the presence of tumor-derived vesicles in the medium, led to increased *O*-GlcNAcylation levels. The elevation of *O*-GlcNAcylation augmented angiogenesis, while its inhibition had the opposite effect. Using immunoprecipitation (IP) coupled with LC-MS/MS analysis, the authors discovered that human cytosolic SerRS was one of the main targets of *O*-GlcNAcylation in endothelial cells and that its increased level corresponded to alterations in GFAT levels (Figure 2B).

The N-terminal TBD of SerRS was shown to be crucial for the interaction with the OGT enzyme, while Ser101 was identified as the primary *O*-GlcNAcylation site of SerRS [96]. Interestingly, Ser101 is a common site for both phosphorylation [92] and glycosylation of SerRS. Ser101 and the surrounding sequence are highly conserved in mammals [65], indicating the importance of its modifications on SerRS regulation and function in these organisms [96]. Using the Ser101Ala mutant and/or an enhancer of *O*-GlcNAcylation, the authors showed that *O*-GlcNAcylation of Ser101 in SerRS, mediated by elevated GFAT, improved the angiogenic activity of endothelial cells. *O*-GlcNAcylation at Ser101 decreased the stability of SerRS due to higher level of interaction with ubiquitin when SerRS is *O*-GlcNAcylated, leading to proteasome-mediated degradation (Figure 2B). Moreover, stimulating *O*-GlcNAcylation reduced SerRS level in the nucleus, while *O*-GlcNAcylation inhibition reversed the effect [96]. The explanation of this effect includes yet another protein interacting with SerRS—importin α5, an adaptor protein for the nuclear receptor importin β (Figure 2B). Mutations or deletions of the nuclear localization signal in the UNE-S domain disrupted the interaction of SerRS with importin α5 and increased the level of SerRS in the cytosol. A similar effect was observed by silencing the gene for importin α5, but also by the induction of *O*-GlcNAcylation. Further experiments showed that SerRS attenuated the binding of transcription factors transcription factor AP-2-alpha (TFAP2A), specificity protein 1 (SP1), c-Myc, and early growth response protein 1 (EGR1) to the GC-rich region of the *VEGFA* proximal promoter, while *O*-GlcNAcylation of SerRS increased their binding. The authors proposed that SerRS competes with these transcription factors for the *VEGFA* promoter, which decreases *VEGFA* expression. The lack of SerRS in the nucleus due to *O*-GlcNAcylation disables it from performing its antiangiogenic function, leading to an increase in *VEGFA* transcription and therefore an increase in angiogenic activities [96].

To validate the results in vivo, it was shown that knockout of GFAT in xenograft nude mouse models of bladder cancer attenuated tumor growth and the tumor endothelial cells contained very little *O*-GlcNAcylated SerRS. At the same time, hyper-*O*-GlcNAcylation in endothelial cells, induced by the GFAT delivered in extracellular vesicles, facilitated SerRS degradation and hindered the repression of *VEGFA* expression. Similarly, bladder endothelial cells isolated from GFAT knockout mice (GFAT1^−^/^−^) showed lower SerRS *O*-GlcNAcylation levels, and consequently higher SerRS and lower *VEGFA* protein levels compared to the wild-type mice [96].

Altogether, the study showed that a lack of nutrients caused tumor cells to excrete extracellular vesicles containing GFAT, which altered the glucose metabolism in the surrounding endothelial cells. The targeted endothelial cells produced *O*-GlcNAc and transferred it to Ser101 of SerRS. *O*-GlcNAcylation reduced SerRS nuclear localization and promoted its proteasomal degradation. This impeded SerRS function and repressed *VEGFA* expression, leading to angiogenesis (Figure 2B) [96]. The discovered connection could have repercussions not only on cancer research but also on diabetes and cardiovascular disease research, as these diseases also develop endothelial dysfunction in connection to elevated protein *O*-GlcNAcylation levels [97].

## 5. Interaction of Human SerRS with Telomeric DNA and Telomere-Binding POT1 Protein Regulates Telomere Length and Senescence

The life cycle of human somatic cells inherently includes aging as a transition from mitotically active cells to growth arrest in the senescence state. The number of possible somatic cell divisions is limited by the length of telomeres, the terminal chromosomal ”caps“ that become shorter with each division [98]. In stem cells and cancer cells, telomerase, a specialized reverse transcriptase, maintains telomere length by adding telomeric repeats to chromosome ends during cell division [99]. Another defense mechanism against damaging telomeric DNA includes the formation of a shelterin complex comprised of six proteins (telomeric repeat-binding factor 1 (TRF1), TRF2, TRF1- and TRF2-interacting nuclear protein 2 (TIN2), repressor-activator protein 1 (RAP1), protection of telomere 1 (POT1), TPP1) [100,101]. Deregulation of telomere-length maintenance has been observed in many physiological and pathological processes, including cancer and aging. Interestingly, a new non-canonical role of human cytosolic SerRS, localized in the nucleus, in controlling telomere length was recently uncovered (Figure 2A) [102]. Overexpression of SerRS resulted in telomere shortening and increased the levels of senescence molecular markers. Experiments with tumor xenografts overexpressing SerRS in immunocompromised mice confirmed a senescence pattern and arrest in tumor development by growth inhibition, indicating that SerRS may function as a tumor suppressor. Once SerRS enters the nucleus, in addition to binding to the *VEGFA* promoter [83,88], it also binds telomeres through direct interaction with telomeric DNA repeats [102]. In a high-throughput protein–protein interaction screen, SerRS was identified as a protein that may interact with the shelterin protein POT1 [103], one of the most important guardians of telomere integrity. POT1 binds a single-stranded telomere 3′ overhang via its two N-terminal oligonucleotide/oligosaccharide binding (OB) fold domains, inhibiting the recruitment of telomerase [104]. The GST pull-down assay confirmed the direct interaction between SerRS and POT1. The C-terminal UNE-S domain of SerRS interacted with the OB1 domain of the POT1 protein. Moreover, overexpression of SerRS significantly increased the tethering of POT1 to telomeres, thus preventing telomerase from efficiently approaching telomeres (Figure 2A). The study indicated SerRS plays an important role in balancing translation and replicative senescence induced by telomere shortening to prevent the malignant proliferation of cells. Intriguingly, a disease-causing mutation in the *SARS1* gene that decreased SerRS aminoacylation activity resulted in cellular senescence, implicating SerRS as a cell growth regulator [73].

## 6. Role of SerRSs in the 3-Methylcytidine Modification of tRNA

tRNAs require extensive posttranscriptional modifications to achieve full activity, and it is becoming increasingly clear that many aspects of the tRNA metabolism and function are regulated through the dynamic introduction and removal of modifications [105]. While 3-methylcytosine in DNA (3meC) is a common and well-studied DNA modification, 3-methylcytosine modification in tRNA (m^3^C) at position 32 of the anticodon loop (m^3^C32) is a constituent of the epitranscriptome, which very recently emerged as an important modulator of cellular functions [106].

In the budding yeast *Saccharomyces cerevisiae*, tRNA m^3^C methyltransferase Trm140 is responsible for m^3^C32 modifications of tRNA^Thr^ and tRNA^Ser^. Notably, yeast SerRS copurifies with Trm140 in an RNA-dependent manner and greatly stimulates m^3^C modification of tRNA^Ser^ in vitro (Figure 3A) [107].

The m^3^C32 modification of tRNA in vertebrates involves multiple enzymes [106]. The four human tRNA m^3^C32 methyltransferases, methyltransferase-like 2A (METTL2A), METTL2B, METTL6, and METTL8, share a conserved C-terminal methyltransferase domain but the N-terminal domain of METTL6 differs significantly compared to the other three methyltransferases [108,109]. METTL2A recognizes and modifies human cytosolic tRNA^Thr^ molecules. The METTL6 N-terminal domain is degenerated and cannot bind human cytosolic tRNA^Ser^ efficiently on its own [110]. It was shown that m^3^C32 decoration of tRNA^Ser^ by METTL6 is completely dependent on the presence of human cytosolic SerRS. Although the Co-IP experiment showed that SerRS could be precipitated with METTL6, SerRS did not interact directly with METTL6. Instead, the interaction was indirect and relied on the presence of tRNA^Ser^ in a tRNA-mediated sandwich (Figure 2A) [108,110]. METTL6 bound the anticodon loop, while SerRS recognized the long variable arm of tRNA^Ser^(GCU). The aminoacylation-defective SerRS mutant stimulated the m^3^C32 activity of METTL6, indicating that tRNA binding, but not aminoacylation by SerRS, contributes to m^3^C32 modification by METTL6. Thus, METTL6 modifies tRNA^Ser^(GCU) with the assistance of SerRS within the METTL6:tRNA^Ser^:SerRS ternary complex.

METTL8 is a nuclear-encoded, mitochondria-localized methyltransferase mediating mitochondrial tRNA^Thr^ and tRNA^Ser^ m^3^C32 modification [111,112,113,114]. Human mitochondrial SerRS was found to interact directly with METTL8 in a tRNA-independent manner, which only slightly stimulated its modification activity (Figure 3D) [112]. METTL8 also interacted with mitochondrial threonyl-tRNA synthetase, which did not stimulate METTL8 activity. However, METTL8 substantially promoted the aminoacylation activities of both aaRSs in vitro, indicating a functional connection between mitochondrial tRNA modification and aminoacylation [109].

The biological function of the tRNA m^3^C32 modification has not yet been fully established. Functional insights into the role of m^3^C32 modification indicate that it influences tRNA structure and fine-tunes translation [106]. The absence of this modification impairs cytoplasmic and mitochondrial translation, leading to functional consequences, such as reduced stem cell pluripotency and impaired mitochondrial function [111,115]. The interdependence of yeast and human methyltransferases and SerRSs suggests that m^3^C32 modification is coordinated by tRNA^Ser^ aminoacylation, which may be a mechanism of ensuring that only properly modified tRNAs enter the translation pool [106].

## 7. The Versatility of Protein–Protein Interactions of SerRS in Prokaryotes and Non-Vertebrate Eukaryotes

AaRSs, including SerRSs, participate in protein–protein interactions in various organisms. Generally, bacterial aaRSs exhibit restrained interactions with other protein partners. However, SerRS was found in a complex alongside several proteins involved in DNA replication using a tandem affinity purification and mass spectrometry (TAP-MS) proteome-wide screen of the bacteria *Mycoplasma pneumoniae* [116]. Interestingly, this complex included putative tRNA/rRNA methyltransferase, implicating the evolutionarily conserved role of SerRS in tRNA modification, as was observed with yeast and human SerRSs.

### 7.1. Interactions of Atypical SerRS with ArgRS and Ribosomal Proteins Optimize Translation in Methanogenic Archaea

In archaea, macromolecular associations of aaRSs were found in *Haloarcula marismortui* [117], *Thermococcus kodakarensis* [118], and *Methanothermobacter thermautotrophicus* [119,120]. Protein–protein interactions between *M. thermautotrophicus* SerRS and other components of the archaeal proteome were analyzed by a yeast two-hybrid screen using an atypical form of SerRS, confined only to methanogenic archaea, as bait. It was shown that SerRS interacted with arginyl-tRNA-synthetase (ArgRS) (Figure 3C) [119]. The SerRS:ArgRS complex suppressed the aminoacylation of naive, unmodified tRNA^Arg^ by ArgRS, likely improving translational accuracy [121]. Robust ArgRS:SerRS interaction promoted serylation and enhanced SerRS:tRNA^Ser^ complex formation at elevated temperature and osmolarity, indicating a role in the thermo- and osmoadaptation mechanisms of thermophilic methanogenic archaea [119]. Both *M. thermautotrophicus* SerRS and ArgRS are associated with the large ribosomal subunit (50S), as determined by ribosome sedimentation assays and a combination of biochemical methods [122]. When SerRS or ArgRS were cross-linked to ribosomes and the samples were analyzed by mass spectrometry, several ribosomal proteins comprising a lateral ribosomal stalk made of P-proteins (P-stalk) and proteins near the P-stalk base at the 50S ribosomal subunit were identified as interactors (Figure 3C) [122]. The P-stalk is a specialized protein–protein interaction platform that recruits translation factors, such as the elongation factors EF-1α and EF-G, to the ribosome [123]. Further characterization by gel-shift assay, microscale thermophoresis (MST), and surface plasmon resonance (SPR) confirmed the existence of multiple contact sites between the two aaRSs and the 50S ribosomal subunit, particularly in the region of the P-stalk and nearby ribosomal proteins. Overrepresented consecutive usage of synonymous codons for the amino acids serine and arginine in the *M. thermautotrophicus* genome indicates that the local concentration of a subset of tRNA isoacceptors near the ribosome is different from the global average in the cytoplasm, possibly because they are retained by an ArgRS:SerRS complex on the ribosome to be recharged and reused [122]. A tRNA recycling mechanism was proposed: the interaction of ribosomal P-stalk proteins and aaRSs could enrich the concentration of aaRS enzymes in the vicinity of the ribosome, facilitating the transfer of their aa-tRNA products to elongation factors and the translating ribosome [122]. Interactions of atypical SerRS with ArgRS and ribosomal proteins probably serve to optimize translation and may contribute to the coordination of cellular processes and the regulation of protein biosynthesis.

### 7.2. Interaction of Yeast Cytosolic SerRS and Peroxin Pex21p Implicates a Connection between Translation and Peroxisome Function

In yeast, a complex formation between cytosolic SerRS and peroxin Pex21p, a protein involved in peroxisome biogenesis [124], was found (Figure 3B). SerRS interacted with peroxin Pex21p via its characteristic C-terminal extension, which is not essential for cellular viability or aminoacylation [61]. SerRS bound both tRNA^Ser^ and Pex21p, forming a ternary complex which modestly increased tRNA binding and serylation [62,125]. Pex21p lacks tRNA recognition capabilities but may induce conformational changes in SerRS to increase the binding of cognate tRNA. So far, no peroxisome-specific function has been described for SerRS. It is noteworthy that Pex21p is overexpressed under oxidative stress conditions, while the level of SerRS is decreased. In yeast, the Pex21p-dependent import of proteins into peroxisomes serves as a controllable pathway to deplete the cytosol from undesired proteins under various stress conditions [124]. Thereby, the interaction between SerRS and Pex21p suggests a potential role of SerRS in the regulation of cellular processes such as oxidative stress and peroxisomal function, which needs to be further investigated.

### 7.3. Heterodimeric Mitochondrial SerRS2 Coordinates the Mitochondrial Translation and Replication in Drosophila via the Interaction of Its SLIMP Subunit and LON Protease

Remarkably, in the insect *Drosophila melanogaster* and probably in all protostome animals, mitochondria-localized SerRS2 is a heterodimer composed of the SerRS2α subunit, associated with the SerRS paralogue SLIMP (Seryl-tRNA synthetase-Like Insect Mitochondrial Protein) that contains mutations in the catalytic site at positions involved in the binding of the seryl-adenylate (Figure 3E) [58]. Interestingly, the N-terminal coiled-coil domain important for tRNA^Ser^ recognition by SerRS was evolutionarily lost in SerRS2α subunit, but retained in the SLIMP subunit. Therefore, the degeneration of the catalytic site pocket in SLIMP is accompanied by the loss of the coiled-coil domain in SerRS2α. Although only one tRNA binding surface is used and the other catalytic site is collapsed, the SerRS2 heterodimer efficiently aminoacylated mitochondrial tRNAs^Ser^ [58]. It has been shown that SLIMP depletion induces a loss in respiratory capacity and a distorted mitochondrial morphology [125]. The *Drosophila* SerRS2α/SLIMP heterodimer is involved in complex regulatory pathways that control mitochondrial DNA replication via the interaction of SLIMP with the substrate-binding domain of the major mitochondrial LON protease (Figure 3E) [126]. This stimulates proteolysis of the mitochondrial DNA-binding protein transcription factor A, mitochondrial (TFAM), thus preventing mitochondrial DNA accumulation. The aminoacylation activity of SerRS2 was not affected by LON, demonstrating that their interaction is compatible with tRNA aminoacylation activity. Additionally, SLIMP was identified as an important factor for cell-cycle progression from the G1 to S phase [127]. Thus, *Drosophila* SerRS2 has evolved an idiosyncratic structure that coordinates mitochondrial translation and replication and participates in cell-cycle control.

### 7.4. Arabidopsis Cytosolic SerRS and BEN1 Indicate a Link between Translation and Brassinosteroid Metabolism

Using yeast two-hybrid analysis, it was discovered that protein BRI1-5 ENHANCED 1 (BEN1), an oxidoreductase involved in the metabolism of brassinosteroid hormones [128], interacts with *Arabidopsis thaliana* cytosolic SerRS (Figure 3F) [67]. It appears that the SerRS:BEN1 complex is specific for the *Brassicaceae* family because BEN1 homologs are found only in these plants. Further investigation, including domain mapping, deletion mutagenesis, and biophysical analysis, revealed that interaction depends on the globular catalytic domain of SerRS and the N-terminal extension of the BEN1 protein [67]. SerRS overexpressor transgenic lines showed that the expression of the BEN1 gene is comparable to its expression in wild-type plants, indicating that SerRS does not play a role in the regulation of BEN1 expression [67]. BEN1 exhibited a modest impact on SerRS aminoacylation activity. Considering that the BEN1 N-terminal extension, which modulates the affinity of the BEN1 binding site for the NADP^+^ cofactor [129], is an interaction domain for SerRS, it is likely that SerRS influences BEN1 activity. Thus, the partnership between SerRS and BEN1 signifies a link between the translation and steroid metabolic pathways within the plant cell. Although *Arabidopsis* SerRS gene expression was not modified during abiotic stress [130], it was shown that the disulfide link protects proteins against oxidative conditions [68] and that SerRS protein level is increased during cadmium exposure [131]. Considering that brassinosteroid hormones regulate plant stress response, SerRS:BEN1 interaction may be important in plant response mechanisms to changing environmental conditions, such as exposure to stressors [67].

## 8. Conclusions

### 8.1. Protein–Protein Interaction Networks of SerRSs

Proteins perform their action in the cell independently or in interaction with other macromolecules, which may lead to the emergence of new properties and functions. Research into the interactome or protein network in the cell provides insight into the physiological complexity of the organism and provides a basis for understanding biological processes. Protein–protein interactions (PPIs) enable SerRSs from various organisms to enhance their primary biological task in translation or to perform new non-canonical functions beyond translation, which significantly expands the range of their action.

Available data on the PPIs of archaeal and plant SerRSs show that the *K*_D_s are in the micromolar range, which indicates their transient regulatory interactions [67,119,122], while PPI-induced posttranslational modifications indicate involvement of human cytosolic SerRS in cell signaling pathways [92,96]. When SerRS interacts with a protein partner in the compartment where translation takes place, this often affects its aminoacylation activity (Figure 3) [62,67,109,119,132]. Several SerRS complexes appear important for the fine-tuning of translation. The complex between *M. thermautotrophicus* SerRS and ArgRS promotes serylation reactions at extreme environmental conditions [119] and suppresses aminoacylation of unmodified tRNA^Arg^ [121]. The interplay of both aaRSs with the ribosomal P-stalk likely promotes tRNA channeling, enabling direct aa-tRNA transfer from aaRS to elongation factor to ribosome without dissociation into the cellular fluid [122]. Therefore, associations of atypical SerRS from methanogenic archaea optimize the translational functionality of aaRS and tRNA molecules. Interactions of yeast and human SerRSs with m^3^C methyltransferases promote tRNA modification or aminoacylation, indicating coordination between these processes in the cytosol and mitochondria [107,109,110]. Interestingly, this interaction appears conserved, from bacteria to humans, which implies its evolutionary importance for the fine-tuning of translation.

Some SerRS interactions indicate species-specific links between translation and other cellular processes (Figure 3). The complex between yeast SerRS and peroxin Pex21p provides a connection between translation and peroxisome function, possibly under stress conditions [62]. The partnership between *Arabidopsis* SerRS and BEN1 suggests a link between translation and brassinosteroid metabolism and may be relevant in the stress response mechanisms in plants of the *Brassicaceae* family [67]. In *Drosophila*, an unusual heterodimeric mitochondrial SerRS (SerRS2α/SLIMP) coordinates mitochondrial translation and replication via interaction with LON protease [126].

#### Human Cytosolic SerRS as a Cellular Hub Protein

Human cytosolic SerRS represents an especially remarkable example, as it binds to various protein and nucleic acid partners (Figure 2). In the cytosol, SerRS performs its canonical function, aminoacylation of tRNA^Ser^ and tRNA^Sec^, preparing substrates for translation. It also enables the recognition of tRNA^Ser^ by the methyltransferase METTL6, which is indispensable for tRNA^Ser^ m^3^C32 modification [110].

When localized in the nucleus, SerRS acts in a repressive manner on *VEGFA* gene expression (Figure 2A). SerRS alone or in complex with other proteins (YY1, SIRT2) competes with activating transcription factors (NFκB1, c-Myc), resulting in a balanced *VEGFA* expression important for proper vascular development and angiogenesis [83,88]. *VEGFA* expression is responsive to various conditions, where SerRS plays a role through its posttranslational modifications (Figure 2B). In hypoxia, ATM/ATR kinases phosphorylate SerRS, which diminishes its binding to the *VEGFA* promoter and enables binding of c-Myc and the hypoxia-specific transcription factor HIF-1α, resulting in enhanced *VEGFA* expression and angiogenesis [92]. The lack of nutrients triggers the metabolic reprogramming of endothelial cells by tumor-derived extracellular vesicles, leading to OGT-catalyzed *O*-GlcNAcylation of SerRS. Glycosylation reduces SerRS nuclear localization, which is dependent on interaction with importin α5, and increases *VEGFA* expression and angiogenesis [96]. Considering that SerRS is a ubiquitous protein present in all cells all the time, SerRS can be regarded as a master negative regulator of *VEGFA* gene expression, being important for proper vasculogenesis as well as for developmental and non-developmental angiogenesis.

Recently, a new SerRS transcriptional repressor activity dependent on posttranslational modification was uncovered [133]. Glucose-dependent SerRS acetylation promoted its translocation to the nucleus, where it suppressed the genes involved in de novo lipid biosynthesis. Histone/protein deacetylases HDAC4 and HDAC5 affected SerRS acetylation levels, and although their direct physical interactions with SerRS have not been investigated, it is very likely that the regulation of SerRS acetylation is mediated through protein–protein interactions. Nuclear localization is important for yet another non-canonical function of SerRS fueled by PPI. SerRS binds telomeric DNA and cooperates with the shelterin protein POT1 to regulate telomere length and cellular senescence (Figure 2A) [102].

The numerous PPIs position human SerRS on the cellular metabolic map as a new hub protein connecting translation with other essential processes such as angiogenesis, lipid biosynthesis, and telomere maintenance.

### 8.2. Connection between PPIs of Human SerRSs and Diseases

Studies of protein interactions result in the identification of protein networks important for cellular functioning, but they are also valuable for biomedical applications. Disruptions of the dynamics and structure of protein networks can lead to disorders and diseases. By identifying important nodes as possible drug targets, knowledge of disease networks can enhance drug design, and productive diagnostic and treatment approaches can be developed.

Pathogenic variants in genes encoding human cytosolic and mitochondrial SerRSs have been shown to cause hereditary disorders. Most of these variants showed reduced serylation activity, which could explain the observed symptoms [71,72,73,74,76,78]. However, for some of the variants, the effect is unknown, which creates the possibility that impaired SerRS PPIs may be the cause of the symptoms that should be taken into account when investigating these disorders [70,75,77,79].

Pathological angiogenesis is a hallmark of many cancers, cardiovascular diseases, diabetic retinopathy, autoimmune diseases, rheumatoid arthritis, atherosclerosis, cerebral ischemia, and delayed wound healing [134]. Starvation and hypoxia strongly drive the activation of endothelial cell functions and provoke continuous neovascularization, and the inhibition of SerRS’s repressive antiangiogenic role has been observed in both of these conditions (Figure 2B) [92,96]. Deregulation of SerRS acetylation led to an obesity-like phenotype in mice and has been linked to abnormally enhanced lipogenesis, which may contribute to the initiation and progression of breast cancer [133]. Deregulation of telomere biology is a contributing factor in many physiological and pathological processes, including aging and cancer, and it has been shown that SerRS is involved in telomere shortening and senescence [102].

Considering that SerRS exhibits a suppressive effect on multiple pathways, such as angiogenesis, lipogenesis, and telomere elongation, all of which contribute to tumor development and progression, SerRS can be regarded as a tumor suppressor and potential anti-tumor drug target or therapeutic agent. Indeed, the expression of a phosphorylation-deficient form of SerRS in mice showed potent antiangiogenic and antitumor effects [92]. A decrease in SerRS *O*-GlcNAcylation attenuated tumor growth in bladder cancer xenograft in mice [96]. Overexpression of SerRS, particularly the acetylation-mimetic SerRS mutant, greatly suppressed the proliferation of breast cancer cells and the growth of breast cancer xenografts in mice [133]. Also, overexpression of SerRS dramatically inhibited the growth of the HeLa cell tumor xenograft and induced the senescence of tumor cells in mice [102]. Interestingly, unlike most aaRSs, a high expression of SerRS is correlated with better clinical outcomes in breast cancer patients [102].

Therefore, antitumor and angiostatic (antiangiogenic) treatments may include the overexpression of SerRS, enhanced import of SerRS into the nucleus, a decrease in SerRS phosphorylation and glycosylation, and an increase in SerRS acetylation. On the other hand, promoting angiogenesis may be valuable in preventing and treating disorders related to hypoxic conditions and ischemia injury, such as ischemic heart disease, cardiovascular diseases, and neurological diseases. Several small molecules, such as all-trans retinoic acid, emodin, and the isoflavone derivative 3-(4-methoxyphenyl) quinolin-4(1H)-one (MEQ), have been identified as inducers of SerRS expression [135,136,137]. A patent (no. WO2018035041A1) proposed the administration of a phosphorylation-deficient SerRS mutant to reduce angiogenesis and a phosphorylation-mimetic mutant to induce it. It is essential that potential treatments do not affect canonical SerRS activity. Considering that SerRS nuclear localization appears not to interfere with its aminoacylation function in translation, focusing on nuclear SerRS functions may hold great promise in the treatment of SerRS-linked diseases.

### 8.3. The Interaction Domains of SerRSs

Although SerRS kept its basic two-domain (TBD and CD) organization during its evolution, it was also amenable to evolutionary inventions through additional extensions and insertions appearing in eukaryotic cytosolic and mammalian mitochondrial SerRSs (Figure 1) [9,56,61,65]. Plant and atypical archaeal SerRSs interact with their respective partners (BEN1, ArgRS) through the catalytic domain (Figure 3) [67,119]. In the case of yeast SerRS and Pex21p, the main interaction domain is the basic C-terminal extension specific for eukaryotes (Figure 3) [61,62]. The vertebrate C-terminal extension, the UNE-S domain, is longer and contains NLS, which enables localization in the nucleus where human SerRS exerts its non-canonical activities [63]. All domains of human SerRS are used in PPIs: TBD is important for interaction with OGT [96], CD for interaction with SIRT2 and YY1 [83,88], and UNE-S for interaction with POT1 (Figure 2) [102]. Human SerRS interacts not only with tRNA^Ser^ and tRNA^Sec^ but also with DNA, such as the *cis*-regulatory promoter elements of the *VEGFA* gene and telomeric repeats [83,96,102], and specific mRNAs [50], indicating its remarkable plasticity and adaptability towards various interaction partners. Similar versatility exhibits SerRS from methanogenic archaea, which interacts with ArgRS and several ribosomal proteins of the P-stalk (Figure 3C) [122].

Structural information on SerRS protein complexes is completely lacking. We still do not entirely understand how SerRS domains are discriminated against in vivo and avoid competition for their ligands, achieving the specificity required for the assembly of complexes and the biological processes that they control. It would be interesting to unveil the exact molecular contacts between SerRSs and their protein partners (and nucleic acids) and decipher how the SerRS structure adjusts to diverse interacting factors.

### 8.4. Outlook

This comprehensive review shows that SerRSs from various organisms engage in protein–protein interactions with diverse proteins. Apart from their conventional task in cytosolic and organellar translation and association with tRNA^Ser^ and tRNA^Sec^, SerRSs interact with other cellular factors, forming protein or nucleoprotein complexes that broaden the range of SerRS functions. Studies across multiple species show that SerRS protein partners vary greatly, depending on the species, and that interactions are, in general, not evolutionarily conserved. In methanogenic archaea, yeast, and plant SerRS, interactions affect serylation and likely fine-tune the translation, but also indicate involvement of SerRS in stress responses, which requires further investigation [62,67,122]. In insect mitochondria, SerRS is involved in the coordination of replication and translation [126].

Human cytosolic SerRS, which is also targeted to the nucleus, is involved in many PPIs, which makes it a multifaceted regulator in fundamental biological processes such as translation, vascular development, postdevelopmental angiogenesis, lipogenesis, and telomere-length maintenance [83,88,102,133]. Conditions in the extracellular environment, such as changes in oxygen and nutrient concentration, affect SerRS posttranslational modifications that regulate downstream processes, making SerRS a cellular hub protein that connects outside cues to intracellular functions [92,96]. As an antitumor and antiangiogenic factor, it appears to be a promising drug target and therapeutic agent for treating cancer, cardiovascular diseases, and possibly obesity and aging.

Future research is needed to explore the interactions of SerRSs and other factors in the control of multiple cellular pathways and to determine how aberrant or disrupted interactions can lead to hereditary disorders and multigenic diseases. An analysis of these protein networks may lead to the discovery of novel higher-order structures. As a result, it may offer a chance to understand how intricate biological mechanisms and changes in SerRS-linked processes impact elaborate diseases like angiogenesis-related disorders and cancer. These protein relationships may point to a novel approach in the development of molecular therapies. Because complex multigenic diseases, as well as genetic diseases, require new, effective diagnoses and therapies, we anticipate that such a systemic point of view will be applicable to these conditions as well as present new opportunities for improving our understanding of the mechanisms underlying these diseases.

## Data Availability

Data sharing is not applicable to this article.
